# Stressors, Emotions and Eating: Evidence for Time‐Pressure‐Driven Snacking Rather Than Emotional Eating

**DOI:** 10.1002/smi.70143

**Published:** 2026-01-23

**Authors:** Christoph Bamberg, Jens Blechert, Julia Reichenberger

**Affiliations:** ^1^ Department of Psychology Paris‐Lodron‐University of Salzburg Centre for Cognitive Neuroscience Salzburg Austria; ^2^ Department of Psychology Ludwig‐Maximilians‐University Munich Munich Germany

**Keywords:** affect, ambulatory assessment, ecological momentary assessment, food, health, intake

## Abstract

Associations between stress, emotions and unhealthy eating have been reported, yet findings remain inconsistent. Stress eating and emotional eating have frequently been used interchangeably despite potential differences regarding physiological and psychological explanations. To aid in disentangling the shared and unique effects of stressors and emotions, we examined their associations with distinct aspects of eating behaviour (food craving, quantity, healthiness and snacking) in daily life in an experience‐sampling study. Participants (*N* = 64) responded to six daily smartphone prompts over 4 weeks, reporting stressors, multidimensional emotional states (valence, arousal, calmness) and eating behaviour. Bayesian multilevel models revealed that general stressors were unrelated to snacking (OR = 1.07, 90% CI = [0.87, 1.32]), but episodes of time pressure increased snacking likelihood (OR = 1.34, 90% CI = [1.01, 1.75]) while being associated with lower food quantity (*B* = −2.45, 90% CI = [−4.07, −0.82]). Arousal was associated with more food intake (*B* = 0.04, 90% CI = [0.001, 0.087]), while there was no other emotional state—eating association. Together, these results indicate that changes in snacking behaviour are more strongly linked to specific situational constraints, such as time pressure, than to momentary emotional fluctuations. Emotional eating, operationalised as emotion‐driven snacking independent of stressors, received little support in this sample. These findings suggest that interventions may benefit from targeting situational constraints, such as time pressure.

## 1 Introduction

An emotional argument with a partner may drive us to seek solace in ice‐cream. A time‐pressured day might force us to gobble down an unhealthy, quick snack. These examples illustrate the constructs ‘emotional eating’ and ‘stress eating’ (see below). Problematic about these eating behaviours is that the consumption of calorie‐dense, high‐fat and high‐sugar foods is more likely which may contribute to a less healthy diet (Hess et al. 2016). Over time, this can lead to substantial negative health outcomes such as overweight and obesity (Mattes 2018; Njike et al. 2016; Skoczek‐Rubińska and Bajerska 2021).

Emotional eating is defined as eating behaviour that is precipitated by emotions (either negative or positive). Importantly, individuals differ substantially in how their eating behaviour is affected by internal emotional states. Some tend to eat more when in a negative emotional state, while others eat less or show no change (Meule et al. 2018a; Reichenberger et al. 2020). This makes emotions potentially a more complex factor in eating than hunger or enjoyment, which both generally predict greater consumption more consistently (Beaulieu and Blundell 2021). Emotions can be characterised along multiple dimensions, including valence, energetic arousal, and calmness (Wilhelm and Schoebi 2007). These dimensions may show distinct associations with eating behaviour, indicating that emotional eating is not a unitary construct.

**FIGURE 1:**
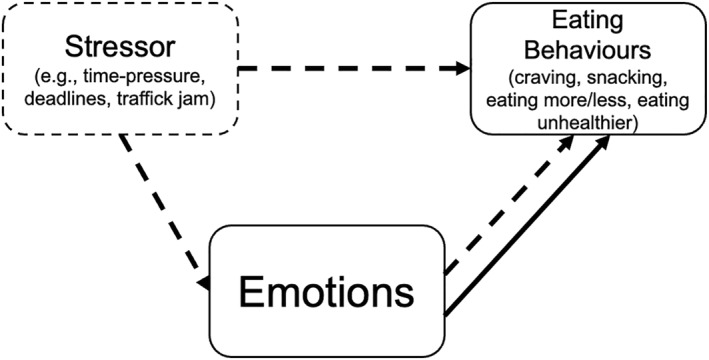
Conceptual relation between stressors, emotions and eating. Conceptual representation of the associations examined between stressors, emotions, and eating behaviour. The effect of stressors on eating is represented by dashed lines, implying a direct relation between stressors and eating (e.g., through time pressure) as well as an indirect relation through effects of stressors on emotions. Emotions also potentially directly affects eating, represented by the solid line. This model implies the associations tested in this study.

At the same time, emotional states rarely occur in isolation but are often embedded in broader situational contexts, such as stressors. Daily stressors—e.g., arguments, deadlines, traffic jams, bad weather or unexpected demands—are ubiquitous and, although individually fleeting, their cumulative burden can exceed coping resources and undermine long‐term health behaviour (Inauen et al. 2025; O’Connor et al. 2021). According to theories (e.g., Lazarus and Folkman 1984), stressors may be appraised and potentially result in stress experiences and related negative emotions. Hence, as can be seen in Figure 1, we propose that stressors may influence eating indirectly via emotional experience, but they may also affect eating more directly without emotion activation (e.g., through imposing time constraints for meal planning or preparation). Lack of meal preparation almost always leads to less healthy choices, for example, snacks, processed foods or pre‐cooked or fast meals (Mills et al. 2017).

Up to now, the terms ‘emotional eating’ and ‘stress eating’ have been used interchangeably and different concepts (emotion, stress, stressor, etc) have been intermingled. Similarly, most studies have not simultaneously modelled stressors and emotions, leaving it unclear whether negative emotions drive unhealthy eating in their own right or are a consequence of a stressor occurrence. Moreover, stress‐eating and emotional‐eating research has evolved in separate tracks regarding methodology and theories: Stress‐eating work centres on physiological explanations (e.g., cortisol), while emotional‐eating studies focus on more psychological explanations such as affect regulation or restraint. Because each uses its own terms and measures—with stress research often ignoring discrete emotions and emotional‐eating research rarely tracking stress biomarkers—their commonalities remain underexplored. It is therefore important to consider the stressor‐emotion‐eating sequence in the same study and explore whether different subcategories of stressors (e.g., interpersonal stressors, time pressure) might exhibit distinct pathways.

On the side of the dependent variables, various eating behaviours (e.g., calories, healthy eating) and prior appetitive experiences (e.g., food craving) have been used (see below for specific findings). Food craving is a proximal indicator of unhealthy eating that captures the motivational pull of tempting (rather high‐caloric) foods (Aulbach, Bamberg, Reichenberger, Arend, and Blechert 2025a; Reichenberger, Richard, et al. 2018b). Food craving is independent from actual availability of foods and can also arise from affective, situational, or physiological factors, making it a useful target for both emotional and stress eating research. However, food craving may not ultimately lead to food intake (e.g., A. J. Hill 2007), so that adding actual eating behaviour might be worthwhile. Hence, the present study uses both food craving (i.e., appetitive experience) and snacking, healthy eating, eating amount (i.e., actual eating behaviour) as dependent variables.

Prior research has already shown the influence of stressors and emotions independently but also in concurrence on eating behaviour: Daily stress experience was associated with elevated cravings (Debeuf et al. 2018; Dicker‐Oren et al. 2024; Groesz et al. 2012), unhealthier food intake (Cleobury and Tapper 2014; Groesz et al. 2012; D. Hill et al. 2022) and snack food frequency (Cleobury and Tapper 2014). In contrast, research also showed that stress can reduce taste‐based eating (Reichenberger, Kuppens, et al. 2018; Reichenberger, Richard, et al. 2018) and potentially the direction of the stressor‐eating association depends on the nature of the stressor (e.g., interpersonal stressors increasing while physical stressors decreasing snacking; O’Connor et al. 2008). Alternatively, the stressor‐eating associations may partly reflect the emotional appraisal of the stressor (indirect path, Figure 1). For instance, stressors led to binge eating only when triggering negative affect in one clinical study (Goldschmidt et al. 2014). Findings on emotion‐driven eating in nonclinical samples are mixed: negative affect reliably precedes binge‐eating episodes in disordered populations (Arend et al. 2024; Haedt‐Matt and Keel 2011; Reichenberger et al. 2020), whereas in healthy individuals it sometimes coincides with reduced intake (Wouters et al. 2018), shows no significant link to eating (Schultchen et al. 2019; Zenk et al. 2014) or depends on inter‐individual differences (Reichenberger, Kuppens, et al. 2018; Reichenberger, Richard, et al. 2018). Furthermore, a collated analysis across samples found only boredom to predict snacking (Aulbach, Bamberg, Reichenberger, Arend, and Blechert 2025b) suggesting emotion specificity. Together, these findings highlight that stressors and emotions are often examined in isolation, making it difficult to determine whether observed eating effects reflect ‘pure’ stressor effects or affective processes of the stressors.

To disentangle the respective shared and unique contributions of stressors and emotions to snacking, we conducted an experience sampling study, collecting intensive longitudinal data over 4 weeks on stressors, emotions, and eating behaviour in young adults. These subjective phenomena are best captured in real‐life contexts in free‐living individuals with variable daily routines and fluctuating demands. Conceptually, we treat stressors as exogenous events that may induce changes to emotional states (which in turn affect eating; indirect path, Figure 1) but also might directly affect eating behaviour (i.e., direct path, Figure 1). Thus, emotions may influence eating behaviour both as a consequence of stressors but also through other stressor‐unrelated affective fluctuations (e.g., watching a sad movie, reading an infuriating social media comment). Accordingly, when analysing stressor–snacking links, we do not control for emotional states; when analysing emotion–snacking links, we adjust for concurrent stressors to isolate emotions' unique effect (see Figure 1). We use Bayesian statistics, since from a Bayesian perspective, jointly modelling stressors and emotions allows null findings to be interpreted as informative. When credible intervals concentrate around null values, this constitutes evidence against meaningful emotion‐ or stress‐driven eating effects rather than merely a failure to detect them.

## 2 Methods

### 2.1 Participants

We recruited participants into the study via social media, flyers on university campus and the city of Salzburg and a pool of psychology students from the university of Salzburg as a convenience sample. Study advertisements emphasised the possibility to learn about one's individual stress and emotional eating tendencies. Individuals were enrolled between January and May 2025. Inclusion criteria were age 18–60 years, BMI 18.5–30 kg/m^2^, and no current or past eating disorder. Participants were selected to represent healthy young to middle‐aged adults able to complete intensive smartphone‐based sampling. Of the 71 enrolled individuals, 64 provided sufficient data for analysis. The final sample consisted mostly of young female adults with BMIs in the healthy range (details are provided in the Demographics section below). Participants received up to 50€ or study participation credits (for psychology students) as remuneration, depending on their response rate as well as a summary of their responses. The study was approved by the ethics committee of the University of Salzburg (approval number GZ 36/2023) and accords to the Helsinki declaration. All participants signed an informed consent form.

### 2.2 Design

The recruitment, participant interaction and data collection of this experience sampling study was conducted entirely remotely. Participants answered preliminary trait and demographics questionnaires. They were then instructed in a video‐call with the experimenters how to use the m‐Path app with which they answered the smartphone questions for this study (Mestdagh et al. 2023). We explained each smartphone question and the concept it was supposed to measure, especially focussing on ambiguous items like craving, healthiness and the specific stressor categories with a standardised script. Participants responded six times daily for 4 weeks. We used a signal‐contingent sampling scheme with fixed intervals and the same questions on each day. The daily prompts were sent at 08:30 a.m., 11:00 a.m., 01:30 p.m., 04:00 p.m., 06:30 p.m., 09:30 PM. The first questionnaire was available between 08:30 a.m. and 10:00 a.m., the last between 09:30 p.m. and 12:00 a.m. each day. For the remaining four prompts, participants were able to open the questionnaire within 60 min. All questions were closed‐form multiple choice, single choice, time (hours and minutes) or slider questions (from 0 to 100, starting at 50, anchors on both sides). To ensure data quality, only participants with a response rate above 50% for all prompts were included in the analysis. Furthermore, the response rate was monitored throughout the 4 weeks of the study. If it fell below 70% in the first or second week, participants were contacted via e‐mail reminding them to respond more.

### 2.3 Experience Sampling Questions

Participants indicated stressors by selecting one or more of these categories: argument/conflict, work/school/university problem, financial issue, health issue/accident, event affecting someone else, time pressure, worrying/brooding, or no stressor. Each of these categories has precedent in experience sampling research. For example, items such as work/school/university problems, health issues or accidents and interpersonal conflict have been included in daily stressor checklists (Koffer et al. 2016; Neupane et al. 2025). Time pressure and financial issues likewise appear in experience sampling items assessing acute stressors (Inostroza et al. 2024). We added worrying/brooding based on pilot feedback to better reflect participants' lived experiences, though in our analysis we only consider external, event‐based stressors. Emotion states were assessed using bipolar sliders for valence (e.g., ‘satisfied’–‘dissatisfied’) energetic arousal (e.g., ‘energised’–‘depleted’), and calmness (e.g., ‘tired’–‘awake’) based on the mood scale of Wilhelm and Schoebi (2007).^1^

As dependent variables, participants reported the number of snacks and main meals consumed since the last prompt. During onboarding, we defined breakfast, lunch, and dinner as main meals; all other intake counted as snacks. Participants rated how much they ate (‘little’–‘a lot’; referred to as ‘amount’ in the results), how healthy it was (‘not at all’–‘very healthy’) and how strongly they experienced craving (‘no craving’–‘strong craving’). Additional items not analysed here included hunger, sleep quality, and concentration (full list: https://osf.io/bmcfk/).

In each assessment window, stressors and eating behaviour were reported retrospectively (‘since the last prompt’), whereas emotion items captured participants' current state at the moment of responding. Our primary models therefore estimate associations between stressors and eating within the same reporting interval, while relating eating behaviour to emotions from the preceding time point, that is, lagging the emotional states, without assuming a fully specified temporal sequence among all variables.

### 2.4 Trait Questionnaires

Demographics and standardised questionnaires were completed before the smartphone‐based experience sampling period. The Salzburg Emotional Eating Scale, SEES (Meule et al. 2018a) and Salzburg Stress Eating Scale, SSES (Meule et al. 2018b) were administered as measures of potential trait‐level inter‐individual differences. The SEES is a 20‐item scale scored into the subscales angry, sad, anxious, happy eating. The SSES consist of 10 items, aggregated into one score.

### 2.5 Analysis Plan

We applied binomial likelihoods with logit link functions for binary snacking outcomes and normal likelihoods with identity links for continuous outcomes (amount eaten, food healthiness). Craving was modelled using a zero‐inflated beta distribution due to excess zeros (logit link for zero‐inflation, log link for beta). For Craving (measured at time t1), stressors (t1) and emotions (t1) were used while for snacking, eating amount and healthiness (t1), stressors (t1) and emotions (t0) were used to account for the way these variables were measured.

Bayesian models used weakly regularising priors (see Supporting Information S1: Appendix). Models were run with brms (Bürkner 2021) in R (v. 4.5) using the default brms No‐U‐Turn Sampler (NUTS) with four chains and 2000 iterations (half warm‐up). Chains were extended to 4000 iterations if R‐hat exceeded 1.02 or effective sample size dropped below 700 for any population‐level effect. Coefficients are reported as posterior means. As preregistered, effects were considered substantial if the 90% Credible Interval (CI) excluded 1 (logistic models) or 0 (normal models). We use 90% credible intervals to balance Type I and Type II error risks and since there is no inherent justification for the common 95% interval used in frequentist statistics. The 90% interval is suitable for experience sampling studies since point estimates are typically small and the goal is to estimate credible directional trends rather than make strict dichotomous decisions. For transparency, we indicated which results would not be substantial according to a 95% Credible Interval with a ‘+’ after the 90% Credible Interval (e.g., 90% CI = [0.90, 0.98]^+^) in the Results.

To account for individual differences, models included participant‐level grouping. Time‐of‐day was modelled with a within‐day grouping factor, as this explained more variance than day‐level grouping (see Supporting Information S1: Figure A1; we preregistered the day‐level grouping). Continuous emotion predictors were grand‐mean centred at the population level and subject‐mean centred for grouped terms.

We recoded the snacking variable as binary, as only 6% of responses indicated more than one snack. From the stressor items, we created a binary variable for the presence of any stressor and separate variables for each stressor category. Events falling into multiple categories (e.g., ‘argument’ and ‘work‐related problem’) were included in all applicable categories. Two items each were aggregated into the emotion dimensions valence, arousal, calmness according to their established factor structure (Wilhelm and Schoebi 2007). Higher values reflect stronger self‐reported emotional experience for that dimension.

Finally, we conducted two sensitivity analyses. First, we tested prior sensitivity by varying prior scale parameters (see Supporting Information S1: Figure A3–A8). Second, to address the imbalance between frequent no‐stressor and rarer stressor episodes, we ran weighted regressions adjusting for observation frequency (Supporting Information S1: Table A6, Figure A2).

## 3 Results

### 3.1 Demographics

Of the enrolled 71 participants, we excluded four due to a response rate below 50% and three who terminated the study prematurely, leaving *N* = 64. The analysed sample represents mostly female (*f* = 50 (78%), m = 12 (19%), d = 1 (2%)), young (*M* = 25.24 years, SD = 8.15) university students (*N* = 53; employed *N* = 9) with an average BMI in the healthy range (*M* = 22.11 kg/m^2^, SD = 2.33, range = [18.66, 29.02]; 11% of participants in an overweight BMI range (BMI > 24.9 kg/m^2^)).

### 3.2 Craving During Episodes With Stressors and Emotions

We first tested whether stressors or the emotion dimensions valence, arousal, calmness were associated with food craving. Neither emotion dimensions nor stressors showed a substantial relationship with craving (Supporting Information S1: Tables A1 and A2).

### 3.3 Snacking and Consumption (Amount and Healthiness) During Episodes With Stressors

**FIGURE 2:**
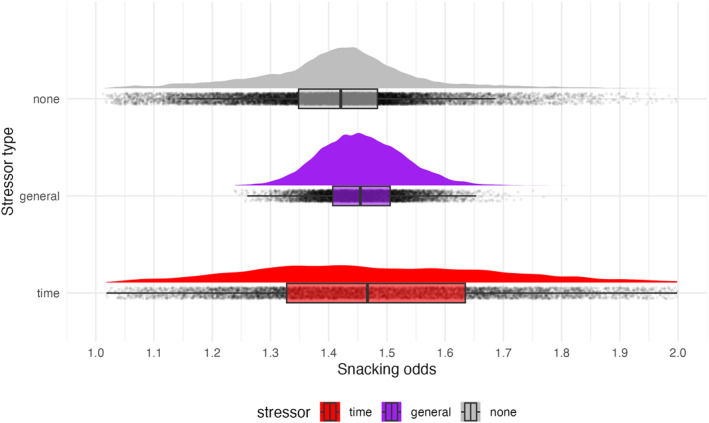
Marginal effects of stressors on snacking odds. The plot shows the marginal (i.e., holding other effects constant) expected predictions for a generic individual drawn from the distribution of random effects (i.e., individual participants). The predictions were generated from separate models for stressor and time‐stressor. The *x*‐axis shows the expected odds of snacking. For each category, the density distribution, boxplot and samples are plotted on the *y*‐axis.

We modelled the odds of snacking with the presence of a stressful event with a multi‐level logistic regression. The probability of snacking without stressors was 28.41% (90% CI = [23.84, 33.64]). It was not substantially higher when a stressor was reported (with 31.10%, on odds scale OR = 1.07, 90% CI = [0.87, 1.32]; Figure 2). Stressors were also not associated with main‐meal consumption (OR = 1.00, 90% CI = [0.89, 1.12]). Notwithstanding, episodes with stressors were associated with lower self‐reported amount of consumed food by 2.78 points on a 100‐point scale (90% CI = [−3.94, −1.59]). We found no association with self‐reported food healthiness OR = 0.25, 90% CI = [−1.31, 1.88].

We further tested whether specific stressor‐characteristics were associated with differences in snacking and consumption (frequencies of stressor categories in Table 1). We found a substantial relation only between time stressors and snacking with 1.34‐fold higher odds of snacking, increasing from 28.41% to 33.00% snacking probability, compared to episodes with no stressor (90% CI = [1.01, 1.75]^+^; Figure 2). We also found an association between episodes with time stressor and a smaller amount of consumed food (OR = −2.45, 90% CI = [−4.07, −0.82]) but no relation with healthiness (OR = 0.92, 90% CI = −1.09, 2.96]).

**TABLE 1:** Frequency of each stressor characteristic (plus worrying).

Stressor characteristic	Count	Percentage
No stressor	7121	67.91
Time stressor	1133	10.81
Worrying	971	9.26
Work/school problem	612	5.83
Health issue	403	3.84
Interpersonal conflict	186	1.77
Financial problem	59	0.56

*Note:* Financial problem was not modelled as a predictor since there were too few episodes with it. Note that these counts are multi‐referenced.

### 3.4 Snacking and Consumption (Amount and Healthiness) in Relation to Emotions

Next, we tested whether momentary emotions could help explain subsequent snack and food consumption. We included stressors as a covariate because any effect of emotions on eating may represent the appraisal of those stressors. As expected, stressor‐presence as well as time‐stressors specifically were associated with lower valence, arousal and calmness (Supporting Information S1: Table A3), supporting the claim that stressors are associated with emotion changes (see Figure 1). In contrast, only arousal was independently associated with the amount of food consumed: a 10 unit increase in momentary arousal was associated with 0.4 more units (0–100 scale) food consumed in the successive time period. There were no further associations between arousal, valence or calmness and the healthiness of consumed food or snacking (Table 2).

**TABLE 2:** Associations between arousal, valence and calmness and eating.

Outcome	Predictor	Coefficient	90% CI
Snacking odds	Valence	1.001	[0.997, 1.004]
	Arousal	0.999	[0.996, 1.002]
	Calmness	1.00	[0.997, 1.003]
Amount	Valence	0.03	[−0.01, 0.06]
	Arousal	0.04	[0.001, 0.087]^+^
	Calmness	−0.001	[−0.03, 0.03]
Healthiness	Valence	−0.01	[−0.06, 0.05]
	Arousal	0.01	[−0.03, 0.06]
	Calmness	0.01	[−0.03, 0.05]

*Note:* The models included stressors as a covariate. 90% CI are the 90% Credible Intervals for the respective posterior. The + symbols indicate that the 95% CI does include 0.

### 3.5 Mediation Model

We also considered emotional states as a mediator in the stressor‐eating relation but did not observe a mediation effect by any emotion dimension for snacking odds, amount, healthiness or craving (Supporting Information S1: Table A4).

### 3.6 Moderating Influence of Trait Questionnaires

There were no substantial moderations of the stressor‐eating (snacking, amount, healthiness, craving) relation or the emotion‐eating relation by the SSES (stress eating style) and SEES (emotional eating style) scales (Supporting Information S1: Table A5) that could explain inter‐individual differences.

## 4 Discussion

The present study intensively sampled individuals' exposure to stressors, emotions and eating behaviour over 4 weeks, providing nuanced insight into daily life. We focused on disentangling the effects of stressors and emotions on various eating behaviours. Experiencing a stressor in general was associated with reduced food intake while time pressure in particular was linked to eating smaller amounts while snacking more. Higher emotional arousal was associated with greater successive food amounts. No other associations emerged between the emotion dimensions (valence, arousal, calmness) and the eating behaviours assessed here (snacking, food healthiness or craving). Moreover, emotional states did not mediate the stressor—eating association. Overall, the Bayesian analysis results provide evidence against sizable emotion‐driven eating behaviour independent of stressors, while highlighting time pressure as a specific situational context associated with altered eating patterns.

Trait eating style questionnaires did not moderate the stressor—or emotion—eating association, suggesting that, in this sample, commonly assumed stable individual differences did not systematically translate into stronger momentary stressor– or emotion–eating associations. This seems in contrast to some previous findings (e.g., Reichenberger et al. 2021, 2018a), but also aligns with others (e.g., Aulbach et al. 2025a, 2025b). Potentially, our overall healthy sample was rather limited regarding emotional and stress‐eating style intensity so that replicating results in more burdened samples (e.g., obesity, eating disorders) might be worthwhile.

### 4.1 Adapting Eating Patterns to Stressful Events

Daily stressors do not affect eating in the same way: Previous work often found interpersonal stressors to be the strongest triggers of craving and dietary lapses, possibly because they have a stronger affective component (Dicker‐Oren et al. 2024; O’Connor et al. 2008). Mixing heterogeneous stressors into a single ‘any stressor’ variable can dilute effects on eating behaviour, especially when episodes span multiple categories (Koffer et al. 2016). Our findings are broadly in line with this: The general stressor factor was not associated with snacking odds or unhealthy intake (although it was associated with less food consumed overall), yet time pressure specifically related to higher snacking and smaller amounts eaten.

This pattern suggests convenience‐ or necessity‐driven adjustment rather than an affect‐driven reaction (O’Connor et al. 2008) and aligns well with previous research indicating more hunger‐based than pleasure‐based eating under time‐pressure (Reichenberger, Kuppens, et al. 2018a). In our study, participants under time stress shifted to snacks—smaller, less preparation‐intensive meals—with no change in self‐rated food healthiness. Furthermore, time pressure may move individuals to consume more calories later in the day (Gill and Panda 2015; Panda 2016). Overall, such an adaptation to time‐pressure may seem like a normative response to situational demands. However, prioritising convenience over quality could incur long‐term health risks, since convenience snacks and meal replacements are typically energy‐dense, higher in refined sugar and saturated fat, lower in fibre and micronutrients (Touvier et al. 2023); sustained substitution of meals with such options degrades diet quality and is linked to higher energy intake and weight gain risk (Mattes 2018; Njike et al. 2016; Skoczek‐Rubińska and Bajerska 2021). This could be a question to specifically ask participants in the future to better understand the impact of such a stressor‐induced shift in eating patterns. By contrast, prior work linking ‘daily hassles’ to both increased snacking and greater high‐sugar, high‐fat intake (Moss et al. 2025; O’Connor et al. 2008) used researcher‐rated food categories, whereas our subjective ratings may understate incidental high‐fat, high‐sugar consumption. Other stressor categories such as interpersonal conflicts, health issues or financial concerns were relatively infrequently reported in our data, which reduces related within‐person variability and limits power to detect associations with eating behaviour. Hence, the absence of an association could be due to their low reporting rate rather than the true absence of behavioural consequences. In general, our findings align with a recent framework review emphasising that associations between stressors and health behaviour in daily life are highly heterogeneous, with studies reporting increased, decreased, or null effects depending on the stress (i.e., stressor characterisation, stress experience) aspect, behaviour examined, timing, and contextual conditions (Inauen et al. 2025).

### 4.2 Only Limited Evidence for Emotional Eating

We found no substantial relations between emotions and eating behaviour, except for a small positive association between arousal and the amount of food consumed. In this context, the isolated association between higher arousal and more food intake observed here seems in line with the psychosomatic account (Bruch 1964) potentially suggesting that physiological arousal might be confused with hunger thereby leading to eating. However, results are also in contrast to previous research suggesting that emotional valence seems an important component (Bongers et al. 2013). Overall, naturalistic evidence for emotional eating in daily life is mixed (Reichenberger et al. 2020), despite experimental and meta‐analytic work suggesting that induced emotional states can influence intake under controlled conditions (Cardi et al. 2015; Devonport et al. 2019). As previously suggested (Aulbach, Bamberg, Reichenberger, Arend, and Blechert 2025b) the emotional eating literature would profit from greater specificity and clearer terminology. The current study takes this approach one step further by focussing on disentangling stressor‐induced emotions versus emotions that might have been activated without any external event. Better understanding the emotion‐related triggers and independent pathways of stressors and emotions might aid in shaping and adapting interventions against unhealthy eating behaviour.

Beyond everyday fluctuations, larger‐scale and sustained stressors may nevertheless alter eating behaviour through their cumulative impact on emotional well‐being. For example, during the COVID‐19 pandemic—a prolonged period of heightened stress and affective strain—several studies reported increases in unhealthy eating patterns, including more snacking and weight gain (Barcın‐Güzeldere and Devrim‐Lanpir 2022; Dolcini et al. 2024; Melamed et al. 2022). Although such macro‐level contexts differ fundamentally from the momentary states captured in experience sampling, these findings suggest that emotion‐related eating effects may become more pronounced under conditions of sustained emotional load, rather than during typical day‐to‐day emotion variation.

### 4.3 Limitations

Our study identified and addressed several challenges for stress‐ and emotional eating research. First, our measures diverged from earlier work: whereas past studies often analysed emotion items in isolation or post hoc aggregates—risking false positives and unstable constructs—we used a validated, multifactor scale. Stressor items, however, were assembled from diverse previous protocols and do not map directly onto any single established checklist, which may limit comparability with other investigations. In addition, all eating variables relied on self‐report, which may introduce under‐reporting of unhealthy or socially undesirable foods and thus attenuate associations, particularly for momentary links between stressors and dietary healthiness.

Second, our sample was drawn from a relatively low‐stress population—university students and professionals—whose everyday stress levels may be lower and less variable than those in more demanding occupations. As a result, our findings may not generalise to groups with higher baseline stress (e.g., clinical populations, groups with low socio‐economic status, refugees) or different age groups, for whom the interplay between stressors, emotions and eating could be quite different. Although the sample did not include individuals with obesity, it did encompass variability in body weight, with 11% of participants in the overweight range. This allows for some heterogeneity in eating behaviour while limiting generalisability to populations with higher BMI. This could be further addressed in the future by experimental ambulatory stress induction (Vikoler et al. 2025) or by recruiting more heterogeneous samples. Moreover, some stressors overlapped, which is to be expected (a conflict with a colleague is both a work and interpersonal stressor), making the distinction between specific stressor‐eating relations challenging but also showing the complexity of daily stressors.

Third, our single‐slider craving measure produced a zero spike and bimodal positive values; zero‐inflated beta models partially addressed this, but latent‐class approaches may better capture low, moderate, and intense craving states (see e.g., Grommisch et al. 2020). More generally, the reliance on self‐reported craving and dietary intake may miss fine‐grained fluctuations that unfold within the reporting interval.

Finally, although most analyses were preregistered, some modelling decisions were informed by empirical characteristics of the data, reflecting a balance between preregistered plans and data‐driven considerations. Moreover, because stressors and eating behaviour were reported retrospectively within the same assessment window, the temporal order of events cannot be fully disentangled. Future studies might profit from using event‐contingent sampling to directly assess stressors and map the subsequent emotion process and eating behaviour.

### 4.4 Conclusion

Our results suggest the following implications for prevention in public health: Targeting contextual constraints, particularly time pressure (and less transient emotions) might be fruitful to reduce unhealthy eating. Even simple psychoeducational approaches—such as increasing awareness of how situational demands shape eating behaviour or improving food and nutrition literacy around convenient yet nutritious snack options—may help individuals anticipate and mitigate risk situations. Such strategies could be implemented both at a universal level and in a more targeted manner for groups frequently exposed to time pressure, including healthcare workers or individuals in high‐demand occupations. By emphasising preparation, accessibility of healthy options, and realistic coping strategies for busy periods, preventive efforts may support healthier eating patterns without framing snacking primarily as a failure of emotional self‐control. For instance, meal‐planning (preparing a healthy meal when you anticipate a busy day) can be further reinforced through implementation intentions (e.g., ‘If I feel pressed for time, then I will eat the salad I've prepared’; as done in O’Connor et al. 2015). Nevertheless, self‐monitoring of emotional states might be worthwhile, although our results suggest a slight shift away from a valence (or potentially also distinct emotions) to an arousal component of emotions.

## Funding

This research was funded in whole or in part by the Austrian Science Fund (FWF) DOI:10.55776/P26_P37164. For open access purposes, the author has applied a CC BY public copyright licence to any author accepted manuscript version arising from this submission.

## Conflicts of Interest

The authors declare no conflicts of interest.

## Supporting information


Supporting Information S1

## Data Availability

Preprocessing and analysis code is available in the OSF study repository (https://osf.io/w6vp8/). Data is not made available at this point since it is used in other still unpublished articles. However, it will be made available individually upon reasonable request.

## Endnotes

^1^
We are aware of the distinction between emotions and mood, however, we wanted to use a daily‐life scale for our experience sampling study that seemed easily applicable, short to answer and psychometrically sound.
