# Supplementary material for: Stressors, Emotions and Eating: Evidence for Time‐Pressure‐Driven Snacking Rather Than Emotional Eating
Source: Stress Health. 2026 Jan 23;42(1):e70143. doi: 10.1002/smi.70143 (PMC12829519; doi:10.1002/smi.70143)

**Additional Methods**

**Priors**

Logistic Model priors: Intercept: Normal(0, 1.5), coefficients: Normal(0, 1.5), varying effects variance: Student-t(3, 0, 2.5), varying effects correlation: LKJ(1).

Normal Model priors: Intercept: Normal(50, 10), Sigma: Half-Cauchy(0.5), coefficients: Normal(0, 5), varying effects variance: Half-Cauchy(0.5), varying effects correlation: LKJ(1).

Zero-inflated Beta Model priors: Intercept: Normal(0, 1.5), coefficients: Normal(0,1.5), varying effects variance: Student-t(3, 0, 2.5), varying effects correlation: LKJ(1), phi and zero-inflated parts: flat default priors.

**Additional Results**

**Grouped terms in multi-level models based on cross-classified model**

Figure A1.

Cross-classified plot for grouping structure


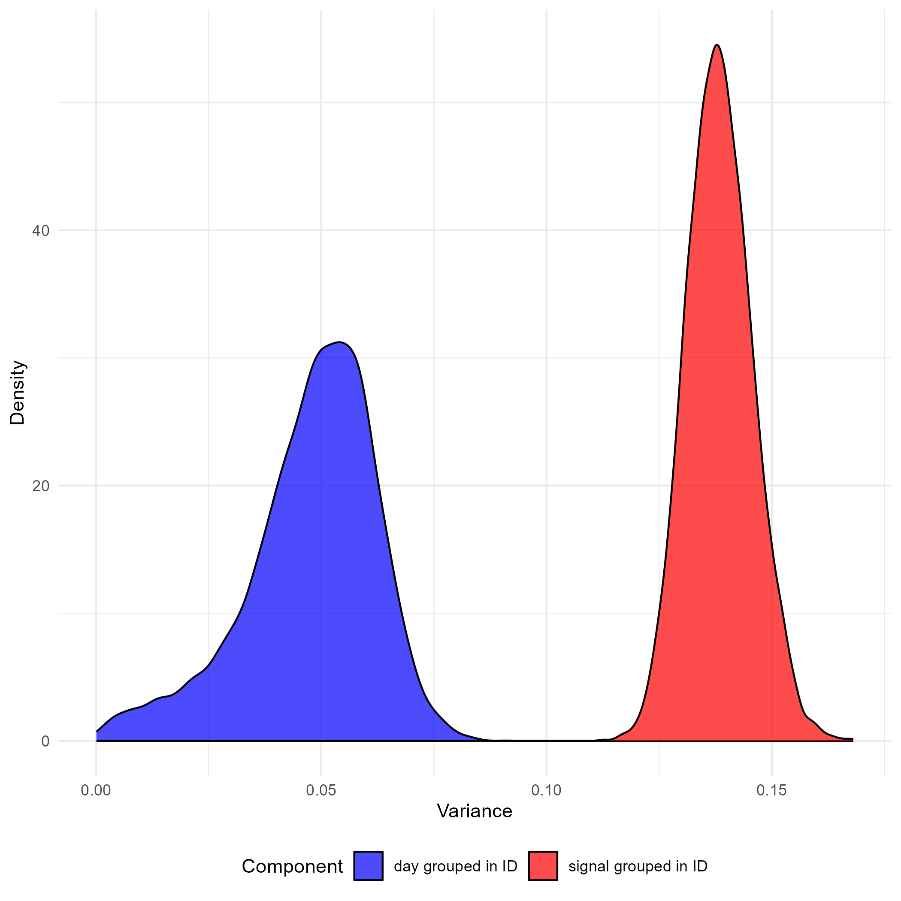


**Craving Results**

**Table A1.**

*Model results mood, controlling for stressors, and craving*

| Mood component | Parameter | Estimate | Est. Error | 90% CI  (Lower) | 90% CI  (Upper) | R̂ | Bulk ESS | Tail ESS |
| --- | --- | --- | --- | --- | --- | --- | --- | --- |
| Valence | Intercept | -0.34 | 0.09 | -0.48 | -0.20 | 1.00 | 982 | 1619 |
|  | Phi Intercept | 1.24 | 0.02 | 1.21 | 1.26 | 1.00 | 5713 | 2593 |
|  | Zi Intercept | -1.02 | 0.03 | -1.06 | -0.98 | 1.00 | 8816 | 3108 |
|  | Valence | -0.00 | 0.00 | -0.00 | -0.00 | 1.00 | 3228 | 3106 |
|  | Stressor | -0.06 | 0.04 | -0.13 | 0.01 | 1.00 | 4857 | 3014 |
| Arousal | Intercept | -0.33 | 0.08 | -0.48 | -0.19 | 1.00 | 1191 | 2090 |
|  | Phi Intercept | 1.24 | 0.02 | 1.21 | 1.27 | 1.00 | 6419 | 2764 |
|  | Zi Intercept | -1.02 | 0.03 | -1.06 | -0.98 | 1.00 | 10514 | 3182 |
|  | Arousal | -0.00 | 0.00 | -0.00 | 0.00 | 1.00 | 2883 | 3142 |
|  | Stressor | -0.05 | 0.05 | -0.13 | 0.02 | 1.00 | 5299 | 3223 |
| Calmness | Intercept | -0.33 | 0.08 | -0.47 | -0.19 | 1.00 | 1477 | 2529 |
|  | Phi Intercept | 1.23 | 0.02 | 1.20 | 1.26 | 1.00 | 5442 | 2743 |
|  | Zi Intercept | -1.02 | 0.03 | -1.06 | -0.98 | 1.00 | 7146 | 2744 |
|  | Calmness | -0.00 | 0.00 | -0.00 | 0.00 | 1.00 | 3042 | 3327 |
|  | Stressor | -0.06 | 0.04 | -0.13 | 0.01 | 1.00 | 4594 | 3187 |

**Table A2.**

*Model results stressor and craving*

| Parameter | Estimate | Est. Error | 90% CI (Lower) | 90% CI (Upper) |
| --- | --- | --- | --- | --- |
| Intercept | -0.41 | 0.08 | -0.57 | -0.25 |
| Phi Intercept | 1.25 | 0.02 | 1.22 | 1.28 |
| Zi Intercept | -2.17 | 0.40 | -2.98 | -1.39 |
| Stressor | -0.06 | 0.04 | -0.14 | 0.02 |

**Stressor-related Additional Findings**

**Table A3.**

*Association between Stressors and Mood*

| Stressor Type | Mood Dimension | Estimate | 90% CI |
| --- | --- | --- | --- |
| General | Valence | -13.21 | [-15.29, -11.11] |
|  | Arousal | -4.88 | [-7.16, -2.56] |
|  | Calmness | -16.70 | [-18.74, -14.64] |
| Time | Valence | -9.09 | [-11.23, -6.95] |
|  | Arousal | -3.46 | [-6.15, -0.84] |
|  | Calmness | -17.00 | [-19.17, -14.79] |

**Moderation and Mediation Analyses**

**Table A4.**

*Stressor—Snacking Association Mediated by Mood Dimensions*

| Direct Effect | Mediator | Estimate | 90% CI |
| --- | --- | --- | --- |
| Stressor—Snacking | Valence | 0.00 | [-0.002, 0.003] |
|  | Arousal | 0.00 | [-0.003, 0.001] |
|  | Calmness | 0.002 | [-0.001, 0.004] |
| Stressor—Amount | Valence | 0.02 | [-0.01, 0.05] |
|  | Arousal | 0.04 | [0.01, 0.06] |
|  | Calmness | -0.01 | [-0.04, 0.02] |
| Stressor—Healthy | Valence | -0.03 | [-0.06, 0.004] |
|  | Arousal | 0.01 | [-0.02, 0.03] |
|  | Calmness | -0.01 | [-0.04,0.20] |
| Stressor—Craving | Valence | 0.002 | [-0.03, 0.03] |
|  | Arousal | -0.01 | [-0.04, 0.01] |
|  | Calmness | 0.03 | [0.004, 0.06] |

*Note:* While two mediations’ 90% CI did not include 0, we refrain from interpreting these associations as substantial, since they are an exploratory analysis and the overall pattern does not show a consistent mediation of the stress—eating relation by mood.

**Table A5.**

*Interaction Coefficients for the Questionnaire Moderators and the Respective Models*

| Scale variable | Model | Coefficient | 90% CI |
| --- | --- | --- | --- |
| SSES score | Stressor-snacking | 0.983 | [0.814, 1.183] |
| SEES sadness | Valence-snacking | 0.999 | [0.995, 1.002] |
|  | Arousal-snacking | 0.997 | [0.994, 1.000] |
|  | Calmness-snacking | 0.997 | [0.994, 1.001] |
| SEES anger | Valence-snacking | 0.998 | [0.994, 1.003] |
|  | Arousal-snacking | 0.998 | [0.993, 1.002] |
|  | Calmness-snacking | 0.997 | [0.992, 1.002] |
| SEES anxiety | Valence-snacking | 0.999 | [0.995, 1.002] |
|  | Arousal-snacking | 0.999 | [0.995, 1.002] |
|  | Calmness-snacking | 0.997 | [0.993, 1.001] |
| SEES happiness | Valence-snacking | 0.999 | [0.993, 1.005] |
|  | Arousal-snacking | 1.002 | [0.997, 1.007] |
|  | Calmness-snacking | 0.999 | [0.994, 0.999] |
| SSES score | Stressor-amount | 0.43 | [-0.97, 1.78] |
| SEES sadness | Valence-amount | -0.003 | [-0.05, 0.04] |
|  | Arousal-amount | 0.01 | [-0.03, 0.06] |
|  | Calmness-amount | 0.01 | [-0.02, 0.05] |
| SEES anger | Valence-amount | -0.01 | [-0.05, 0.04] |
|  | Arousal-amount | -0.04 | [-0.03, 0.06] |
|  | Calmness-amount | 0.01 | [-0.02, 0.05] |
| SEES anxiety | Valence-amount | -0.01 | [-0.06, 0.03] |
|  | Arousal-amount | -0.05 | [-0.1, 0.002] |
|  | Calmness-amount | -0.001 | [-0.001, 0.000] |
| SEES happiness | Valence-amount | 0.04 | [-0.02, 0.11] |
|  | Arousal-amount | 0.04 | [-0.03, 0.11] |
|  | Calmness-amount | -0.001 | [-0.05, 0.05] |
| SSES score | Stressor-healthy | -0.76 | [-2.32, 0.78] |
| SEES sadness | Valence-healthy | 0.002 | [-0.05, 0.05] |
|  | Arousal-healthy | 0.02 | [-0.03, 0.07] |
|  | Calmness-healthy | -0.002 | [-0.04, 0.04] |
| SEES anger | Valence-healthy | 0.02 | [-0.05, 0.08] |
|  | Arousal-healthy | -0.01 | [-0.08, 0.05] |
|  | Calmness-healthy | 0.03 | [-0.03, 0.09] |
| SEES anxiety | Valence-healthy | 0.04 | [-0.02, 0.09] |
|  | Arousal-healthy | 0.02 | [-0.03, 0.08] |
|  | Calmness-healthy | 0.03 | [-0.02, 0.08] |
| SEES happiness | Valence-healthy | -0.08 | [-0.15, 0.001] |
|  | Arousal-healthy | -0.1 | [-0.17, -0.02] |
|  | Calmness-healthy | -0.04 | [-0.1, 0.03] |
| SSES score | Stressor-craving | 2.10 | [0.59, 3.57] |
| SEES sadness | Valence-craving | -0.06 | [-0.10, -0.01] |
|  | Arousal-craving | -0.01 | [-0.05, 0.03] |
|  | Calmness-craving | 0.000 | [-0.04, 0.04] |
| SEES anger | Valence-craving | -0.06 | [-0.12, 0.01] |
|  | Arousal-craving | 0.01 | [-0.05, 0.07] |
|  | Calmness-craving | 0.02 | [-0.04, 0.07] |
| SEES anxiety | Valence-craving | -0.02 | [-0.07, 0.03] |
|  | Arousal-craving | 0.02 | [-0.04, 0.06] |
|  | Calmness-craving | -0.002 | [-0.05, 0.04] |
| SEES happiness | Valence-craving | 0.10 | [0.03, 0.17] |
|  | Arousal-craving | 0.03 | [-0.03, 0.10] |
|  | Calmness-craving | 0.01 | [-0.06, 0.07] |

*Note:* Although a small number of 90% credible intervals exclude the null, we interpret these cautiously in light of the large number of tests and the overall pattern of results, which shows little consistent evidence for associations.

**Sensitivity analyses**

**Weighted regressions for snacking**

Note that participants reported 2.76 times more episodes without stressor than with stressor. To account for this imbalance, we also ran the stressor model weighting each observation by the inverse of their relative frequency. However, this exploratory analysis did not lead to a substantially different outcome.

**Table A6.**

*Model Results of Weighted Regression*

|  | Estimate | Est. Error | l-90% CI | u-90% CI |
| --- | --- | --- | --- | --- |
| Intercept | 0.416 | 1.129 | 0.341 | 0.510 |
| Stressor | 1.026 | 1.135 | 0.833 | 1.263 |

**Figure A2.**

*Comparison Unweighted and Weighted Regression Coefficients*


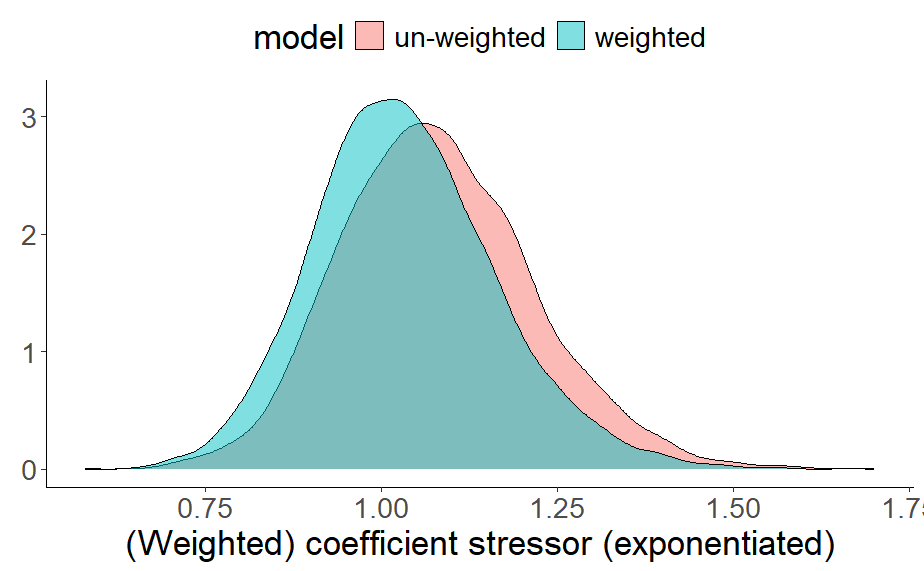


**Prior checks**

We assessed how the chosen prior for the coefficients affects the posterior results by varying the uncertainty in the prior, keeping its location constant at 0.

The plots below show the posterior for the respective model coefficients.

**Figure A3.**

Prior Sensitivity Analysis for the Association between Stress and Snacking


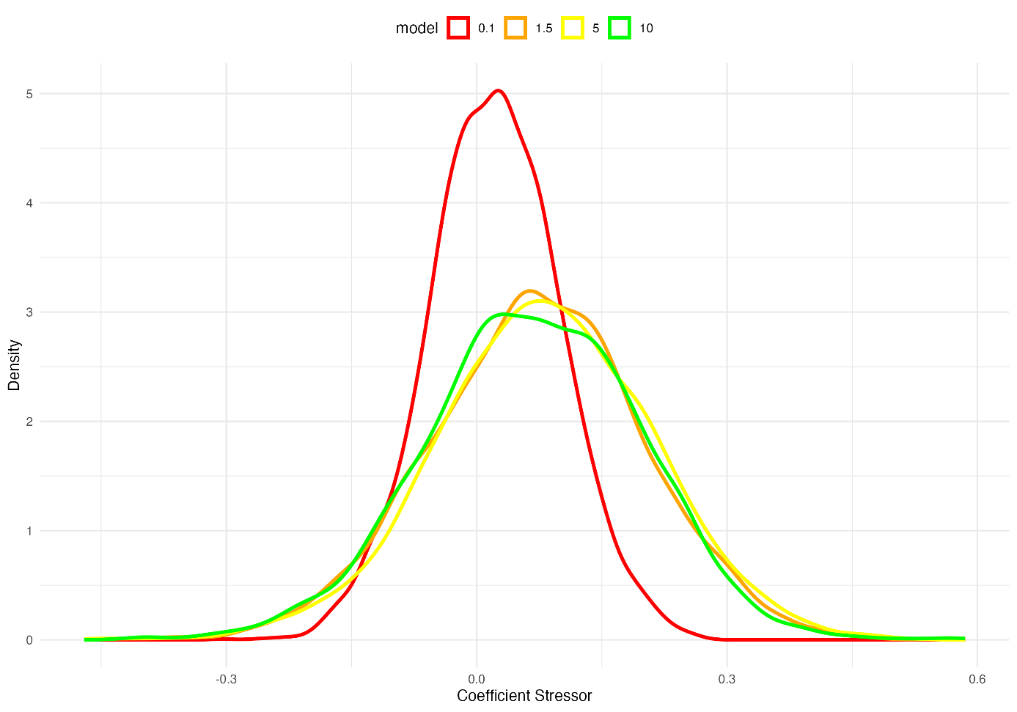


**Figure A4.**

Prior Sensitivity Analysis for the Association between Mood and Snacking


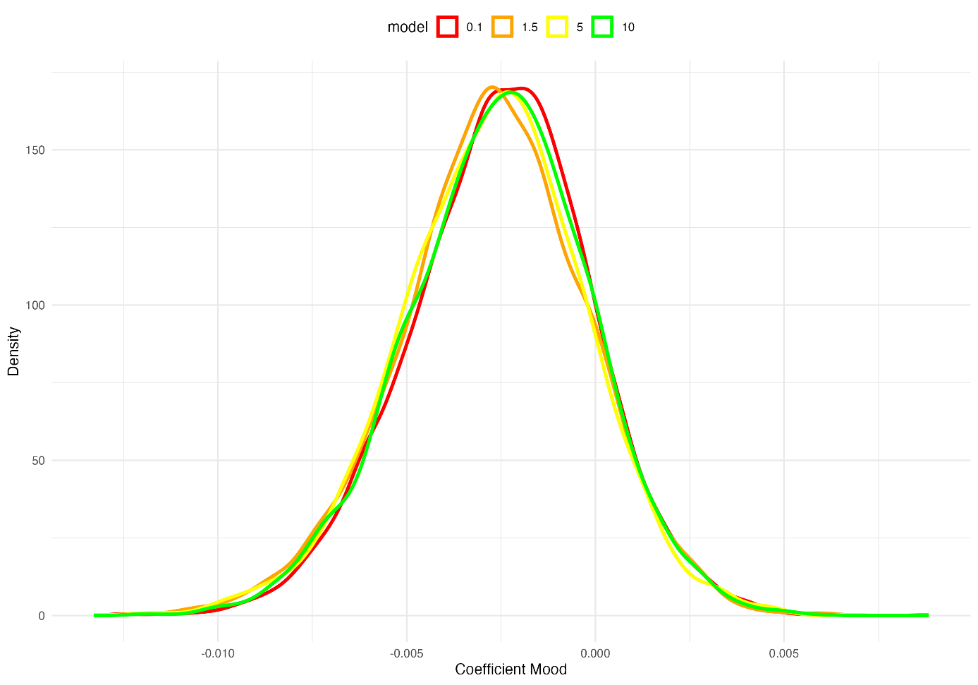


**Figure A5.**

Prior Sensitivity Analysis for the Association between Stress and Healthiness


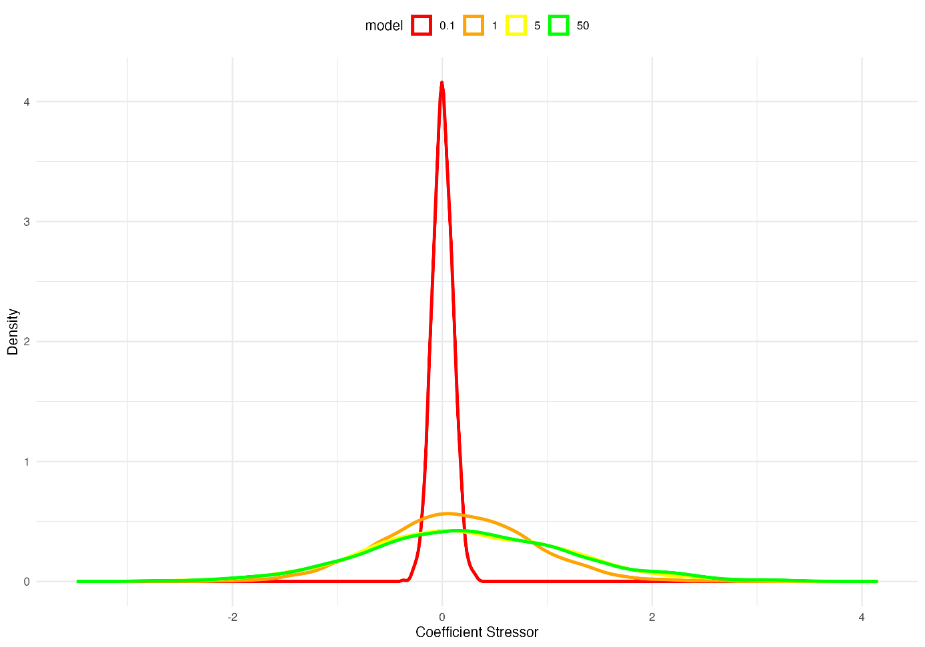


**Figure A6.**

Prior Sensitivity Analysis for the Association between Mood and Healthiness


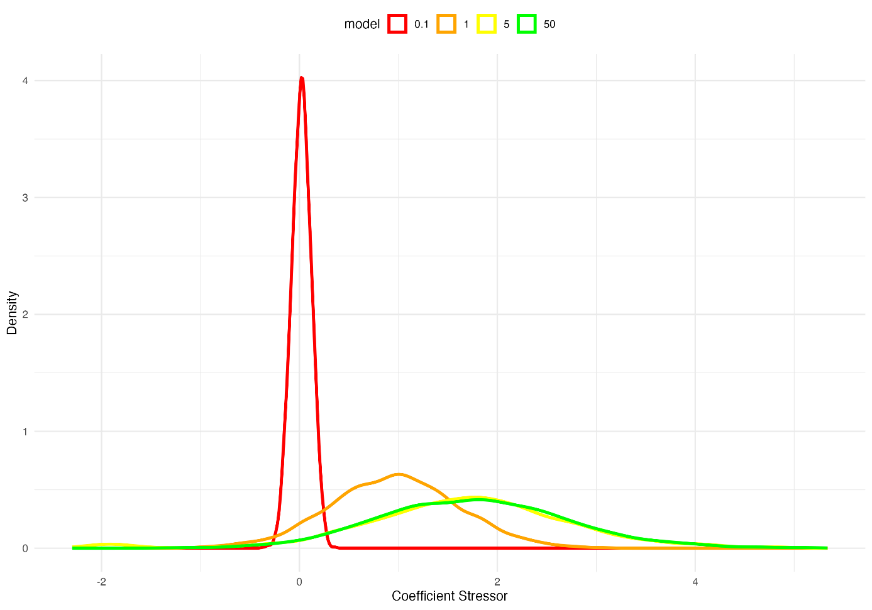


**Figure A7.**

Prior Sensitivity Analysis for the Association between Stress and Amount Eaten


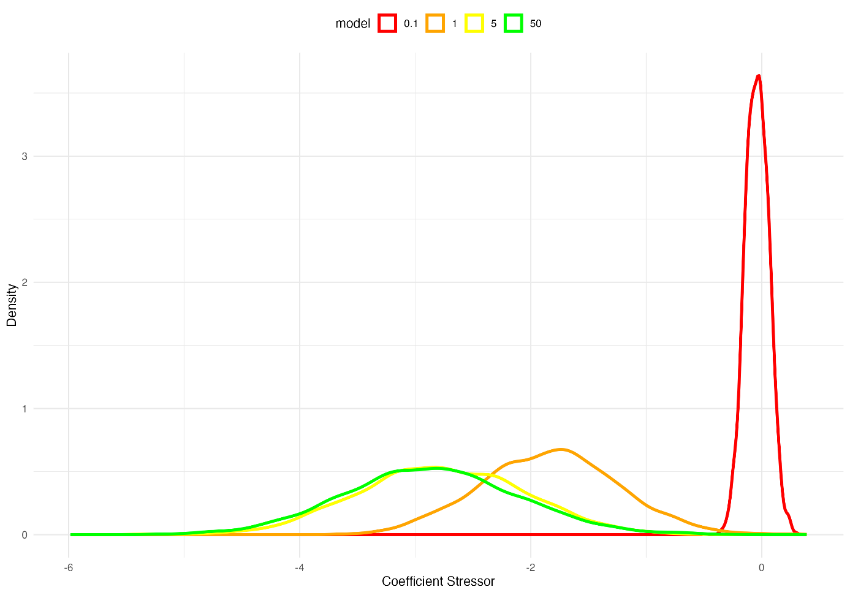


**Figure A8.**

Prior Sensitivity Analysis for the Association between Mood and Amount Eaten


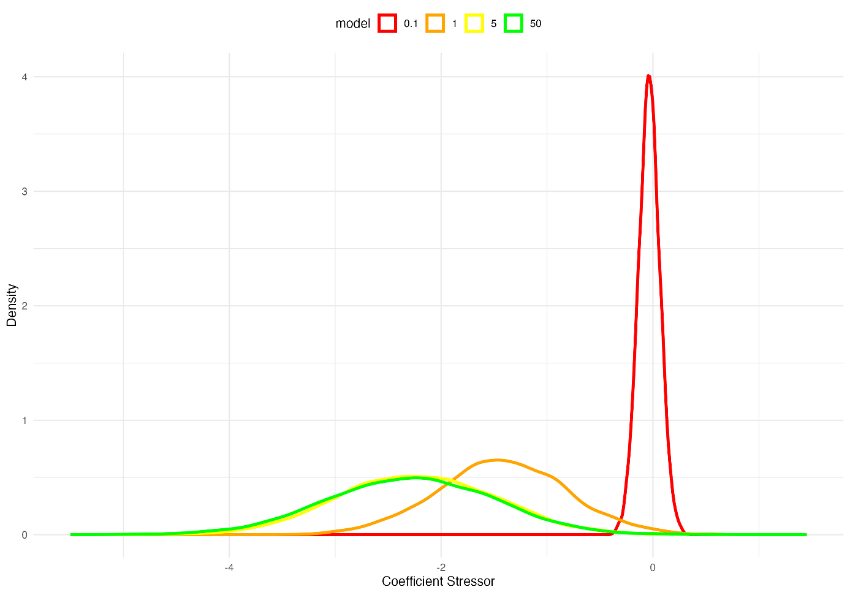

Supplement: Supplementary file 1 — Supporting Information S1 [file SMI-42-e70143-s001.docx]
